# Genetic Diagnosis of Hereditary Hemorrhagic Telangiectasia: Four Novel Pathogenic Variations in Turkish Patients

**DOI:** 10.4274/balkanmedj.galenos.2019.2019.7.2

**Published:** 2019-12-20

**Authors:** Mehmet Baysal, Selma Demir, Elif G. Ümit, Hakan Gürkan, Volkan Baş, Sedanur Karaman Gülsaran, Ufuk Demirci, Hakkı Onur Kırkızlar, Ahmet Muzaffer Demir

**Affiliations:** 1 Department of Hematology, Trakya University School of Medicine, Edirne, Turkey; 2 Department of Medical Genetics, Trakya University School of Medicine, Edirne, Turkey

**Keywords:** ENG mutations, genotype, hereditary hemorrhagic telangiectasia, phenotype

## Abstract

**Aims:**

Hereditary hemorrhagic telangiectasia is an autosomal dominant disorder characterized by telangiectasia, epistaxis, and vascular malformations. Pathogenic mutations were found in *ENG, AVCRL1, SMAD4*, and *GDF* genes. In this study, we present our database of patients with hereditary hemorrhagic telangiectasia regarding the phenotype-genotype relations and discuss two novel ENG gene pathogenic variations in two unrelated families.

**Methods:**

Next Generation Sequencing analysis was performed on the peripheral blood of nine patients with hereditary hemorrhagic telangiectasia in four unrelated families. All patients were diagnosed with hereditary hemorrhagic telangiectasia according to the Curaçao criteria. Data on treatment and screenings of visceral involvement were recorded from files.

**Results:**

We have found a pathogenic variation in either the *ENG* or *ACVRL1* gene in each family. Two novel pathogenic variations in the *ENG* gene, including NM_000118.3 (ENG): c.416delC (p.P139fs*24) and NM_000118.3(ENG): c.1139dupT (p.Leu380PhefsTer16), were found in the same family. The NM_000020.2(ACVRL1): c.1298C>T (p.Pro433Leu) pathogenic variation in the ACVRL1 gene in our first family and a novel heterozygous likely pathogenic NM_000020.2(ACVRL1): c.95T>C (p.Val32Ala) variation was found in our second family. Seven of the nine patients were treated with thalidomide for controlling bleeding episodes. All patients responded to thalidomide. In one patient, the response to thalidomide was lost and switched to bevacizumab.

**Conclusion:**

In hereditary hemorrhagic telangiectasia, certain types of mutations correlate with disease phenotypes and with next generation sequencing methods. New pathogenic variations can be revealed, which might help manage patients with hereditary hemorrhagic telangiectasia.

Hereditary hemorrhagic telangiectasia (HHT), also known as Osler-Rendu-Weber syndrome, is an autosomal dominant disorder with a prevalence of 1/10.000, characterized by angiodysplastic lesions. Expression of the disease is widely variable with an age-related penetrance (1). The diagnosis of hereditary hemorrhagic telangiectasia is made with the Curaçao criteria, which consist of epistaxis, telangiectasia, vascular malformations, and autosomal dominant penetrance. Three criteria are required for the definite diagnosis of hereditary hemorrhagic telangiectasia (2).

The genetic abnormalities of hereditary hemorrhagic telangiectasia are pertinent to the transforming growth factor-beta/bone morphogenetic protein signaling pathway, which is the mainstay of the regulation of various physiological processes, particularly angiogenesis (3).

The *endogolin (ENG)g* (*131195), *activin A receptor type 1 (AVCRL1)* (*601284), *SMAD4 (SMAD family member 4)* (*600993), and *growth differentiation factor 2 (GDF2) *(*605120) genes have been identified as the underlying cause of hereditary hemorrhagic telangiectasia (4). Hereditary hemorrhagic telangiectasia phenotypes are due to mutations in the coding region of the *ENG* and *ACVRL1* genes in the majority of patients (5), whereas *SMAD4* gene mutations occur in 2%-3% of patients (6). Mutations of the *BMP9* gene are rarely reported in patients with hereditary hemorrhagic telangiectasia. Still, mutation may not be found in more than 15% of patients who are clinically diagnosed with hereditary hemorrhagic telangiectasia (7). Patients with *ENG* mutations are classified as HHT1, and patients with *AVCRL1* mutations are classified as HHT2. To date, more than 400 distinct mutations have been reported in the *ENG* gene, including deletions, insertions, duplications, nonsense, and missense mutations, and 246 mutations in the *ACVRL1* gene (7,8,9).

Here we aimed to present the preliminary results of a genetic analysis of hereditary hemorrhagic telangiectasia in our region and report two novel *ENG* variations observed in two unrelated families with hereditary hemorrhagic telangiectasia.

## MATERIALS AND METHODS

### Subjects

Four female and five male patients from 4 unrelated families diagnosed with hereditary hemorrhagic telangiectasia were included in this study. All patients were diagnosed with hereditary hemorrhagic telangiectasia according to the Curaçao criteria. The data on bleeding episodes, the requirement for transfusion and iron replacement, antiangiogenic treatments, and screenings of visceral involvement were recorded from files. Ethical approval was obtained from the local ethics committee, and written informed consent was obtained from all patients (protocol no: TUTF-BAEK 2019/259 date:01.07.2019).

### Genetic analysis

All individuals were subjected to genetic counseling and molecular testing. DNA was isolated from peripheral blood samples of patients by using the EasyOne DNA isolation system (Qiagen, Hilden, Germany). A custom-designed QIAseq Targeted DNA Panel (Qiagen, Hilden, Germany) was used for sequencing the entire coding region of *ACVRL1*, *ADAM17*, *ENG*, *GDF2*, *PTPN14*, *RASA1*, and *SMAD4* genes. QCI analysis (Qiagen, Hilden, Germany) was used to control quality parameters, and Clinical Insight (Qiagen, Hilden, Germany) was used to determine variations. Variants were classified according to ACMG 2015 criteria (10).

## RESULTS

The mean age of the patients was 59 years (29-76). All patients presented and were diagnosed with intractable epistaxis. The mutation screening results and patient demographics are summarized in Table 1. Gastrointestinal bleeding was observed in one patient (patient no: 9), and heavy menstrual bleeding was observed in one patient (patient no: 7).

Our first family has four affected individuals with hereditary hemorrhagic telangiectasia, and all of them have a novel, heterozygous, likely pathogenic NM_000020.2(ACVRL1): c.1298C>T (p.Pro433Leu) variation. Two different missense variations, NM_000020.2:c.1298C>G (p.Pro433Arg) (10) and HGVS NM_000020.2:c.1297C>T(p.Pro433Ser) have previously been reported in the literature for the same codon of the *ACVRL1* gene (11). Three of the four affected members of this family were treated with thalidomide.

The second family has two affected individuals who have a novel, heterozygous, likely pathogenic NM_000020.2(ACVRL1): c.95T>C (p.Val32Ala) variation. Another missense variation; NM_000020.2:c.95T>G (p.Val32Gly), has been reported in the literature for this position on the *ACVRL1* gene (12). Both patients have clinical findings, family history, telengiectasias, and epistaxis episodes. This combination of findings in these patients was treated with thalidomide.

In our third family, we have two affected individuals, and both were affected family members. A 49-year-old and a 76-year-old mother had a novel NM_000118.3(ENG):c.416delC (p.P139fs*24) variation in the *ENG* gene This variation was not reported in the dbSNP or ClinVar and classified as “pathogenic” by our laboratory according to ACMG 2015 criteria (10) (PVS1, PM2, PP3). The bleeding history of the female patient (patient no: 7) consisted of heavy menstrual bleeding alone in her early years that was treated with iron replacement and recurrent severe epistaxis after age 60, which required multiple attempts of surgical and local control. The diagnosis of hereditary hemorrhagic telangiectasia was based on the presence of three of the Curaçao criteria, and her hereditary hemorrhagic telangiectasia Epistaxis severity score was 7.31 (13). To control her epistaxis episodes, oral thalidomide 100 mg/day was initially started. She responded well to thalidomide for a year and reported only trivial episodes of epistaxis without the need for medical care. Her Epistaxis severity score in the first year of treatment decreased to 4.94. Hemoglobin levels were maintained above 10 g/dL. No adverse effects were observed. However, during the second year of thalidomide, epistaxis episodes became more frequent, and her Epistaxis severity score level raised to 7.19. Treatment was switched to bevacizumab. The second member of the same family, the son, reported only trivial epistaxis episodes, which have been managed with local measures.

The proband from the fourth family has a novel NM_000118.3(ENG):c.1139dupT (p.Leu380PhefsTer16) variation in the *ENG* gene. This variation was not reported in the literature or databases before and was classified as “pathogenic” by our laboratory according to ACMG 2015 criteria (PVS1, PM2, PP3) (14). This patient responded to thalidomide treatment.

The initial treatment of patients was local compression, cauterization, and iron supplements, all without a sustainable response. Seven of the nine patients were further treated with thalidomide 100 mg/day orally. İn two patients, no systemic treatment was necessary. The treatment was well tolerated with minor side effects comprising grade one dizziness and nausea, which did not require additional medication (15). Epistaxis episodes decreased in all patients. In one patient, thalidomide was switched to bevacizumab because of clinical effectiveness. The *ACVRL1* mutation was observed in six patients. As categorized according to mutation status, six patients were observed as having HHT2, while three patients were observed as having HHT1. Detailed information regarding hereditary hemorrhagic telangiectasia type and phenotype correlations are provided in Table 2.

## DISCUSSION

In a hypothetical model of hereditary hemorrhagic telangiectasia called a “two-hit model,” it has been suggested that a germline heterozygous mutation in the hereditary hemorrhagic telangiectasia gene may be regarded as the first hit, while inflammation, vascular injury, hypoxia, and angiogenesis may be regarded as the second hit of developing hereditary hemorrhagic telangiectasia. Similar to the multi-hit hypothesis of venous thromboembolism, haploinsufficiency alone caused by mutations may not be enough for the clinical picture; the addition of other processes may explain the “intrafamilial phenotypic variability” of the disease (8,9). As the processes of the second hit may be observed with aging, we may extrapolate that age may also be an additional hit in the variability of phenotype. Supporting this notion, in a retrospective study, epistaxis has been reported as the first symptom, whereas other manifestations related to visceral organs tended to occur at more mature ages explained by the role of inflammation, vascular injury, and aging (16). In the second family of our cohort, a mother and son had the same novel pathogenic variation with different phenotypes. The younger patient had a much milder phenotype, while the older patient had severe and challenging bleeding episodes and a non-sustained response to antiangiogenic treatments.

Endoglin is a 180 kDa transmembrane glycoprotein that functions as a co-receptor for the transforming growth factor-beta receptor complex, which regulates *ALK1* and *SMAD* signaling (4,17). Both truncating and nontruncating mutations in ENG have demonstrated to be associated with HHT1 causing the genes to behave as null alleles, defined as haploinsufficiency. As endothelial cells are depleted with endoglin due to this haploinsufficiency, all physiological processes mediated by transforming growth factor-beta become abnormal, which leads to angiodysplasia (17,18,19). The severity of the phenotype is reported to be different between patients who harbor the *ENG* mutation though no specific variant and is related to a more severe phenotype while in patients with missense mutations, a milder phenotype is reported (10,20,21,22). There were 507 reported variations in the *ENG* gene in the Hereditary Hemorrhagic Telangiectasia Mutation Database of Utah University (23). Our patients’ variation was not reported in this database.

As hereditary hemorrhagic telangiectasia is classified according to mutations of either the *ENG* or *ACVRL1* genes, the phenotype has also been reported to vary in HHT1, and HHT2, such as pulmonary AVMs, vary more commonly in HHT1, while hepatic AVMs vary in HHT2 (20,22). In our study, we observed similar penetrance as pulmonary penetrance in patients with hereditary hemorrhagic telangiectasia and hepatic AVMs in patients with HHT2. None of our patients had cranial AVMs. In our limited analysis; we found more *AVCRL1* mutated patients than *ENG* mutated patients. Diagnosis of hereditary hemorrhagic telangiectasia was made with clinical findings; however, different types of mutations could cause different types of diseases. Therefore, the mutational analysis provides disease classification (HHT1, HHT2, HHT4) and genotype-phenotype correlations. Thalidomide and bevacizumab are the most commonly used agents in hereditary hemorrhagic telangiectasia. Thalidomide is mainly used in multiple myeloma and bevacizumab in several cancer types (24,25,26). The use of both drugs relies on rational logic, but they are not curative treatment options, and there are still large gaps and unmet needs in the treatment of patients with hereditary hemorrhagic telangiectasia. In our limited number of patients with the *ACVRL1* mutation, we observed a positive effect of thalidomide, which should be evaluated with further studies.

This study has two distinct, yet related messages. The first is the existence of four novel pathogenic *ENG* variations, which support the genetic diagnosis of hereditary hemorrhagic telangiectasia within families, and the second message is that further supportive data are needed regarding the contribution of other factors to the phenotype of hereditary hemorrhagic telangiectasia, including age and inflammation.

## Figures and Tables

**Table 1:**
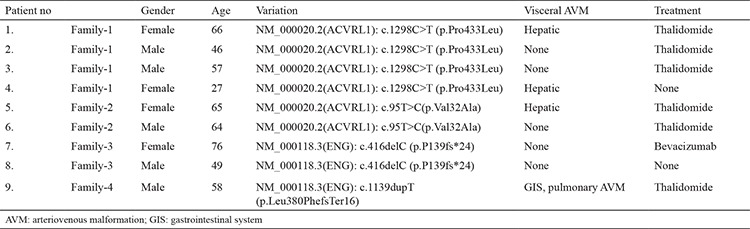
Summary of patient demographic information and mutational analysis

**Table 2:**
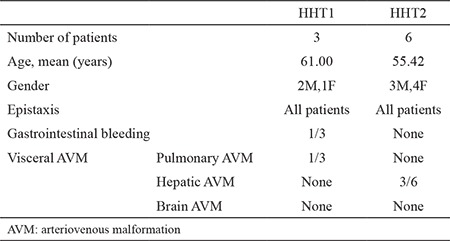
Clinical features and genetic classification
